# Lipid biomarkers and Cancer risk - a population-based prospective cohort study in Taiwan

**DOI:** 10.1186/s12944-021-01570-1

**Published:** 2021-10-10

**Authors:** Yu-Chen Chang, Chien-Ju Lin, Tzu-Lin Yeh, Ming-Chieh Tsai, Le-Yin Hsu, Kuo-Liong Chien, Hsin-Yin Hsu

**Affiliations:** 1 Department of Family Medicine, MacKay Memorial Hospital, No. 92, Section 2, Zhongshan North Road, Taipei City, 10449 Taiwan; 2 The Department of Medicine, MacKay Medical College, No. 46, Sec. 3, Zhongzheng Rd, New Taipei City, 25245 Taiwan; 3 Department of Family Medicine, Hsinchu MacKay Memorial Hospital, No. 690, Section 2, Guangfu Road, East District, Hsinchu City, 30071 Taiwan; 4 Institute of Epidemiology and Preventive Medicine, National Taiwan University, Room 517, No. 17, Xu-Zhou Rd, Taipei City, Taiwan 10055; 5 Department of Endocrinology, Department of Internal Medicine, Mackay Memorial Hospital, Tamsui Branch, 25160 New Taipei City, Taiwan; 6 Department of Internal Medicine, National Taiwan University Hospital, No. 7, Zhongshan S. Rd., Zhongzheng Dist, Taipei City, Taiwan 10002

**Keywords:** Blood lipids, Interval change, cancer risk, Cohort study, Total cholesterol, Low-density lipoprotein cholesterol, Non-high-density lipoprotein cholesterol

## Abstract

**Background:**

Blood lipids are essential components for cellular growth. An inverse association between serum lipid levels and risk of cancer has led to a controversy among previous studies. The aim of this prospective cohort study was to investigate the association between blood lipids change and risk of cancer incidence.

**Methods:**

A cohort of 4130 Taiwanese adults from the Taiwanese Survey on the Prevalence of Hypertension, Hyperglycemia, and Hyperlipidemia database underwent repeated examinations in 2002 and 2007. Six groups were established based on the combined baseline (lower/higher) and interval change (decreasing/stable/increasing) in plasma lipid levels. Multivariable Cox proportional hazard model was used to investigate the relationship between lipids change and all-cause cancer incidence.

**Results:**

Two hundred and forty cancer events developed over a median follow-up of 13.4 years. Comparing these with individuals with decreasing lower-baseline lipid levels, cancer risk reduction was demonstrated in those with increasing lower-baseline total cholesterol (adjusted hazard ratio [aHR], 0.48; 95% confidence interval [CI], 0.27 to 0.85), low-density lipoprotein cholesterol (LDL-C; aHR, 0.56; 95% CI, 0.35 to 0.92), and non–high-density lipoprotein cholesterol (non-HDL-C) (aHR, 0.54; 95% CI, 0.31 to 0.92) levels. A decreased risk for cancer incidence also presented in participants with stable lower-baseline, decreasing and increasing higher-baseline LDL-C levels, and with decreasing and stable higher-baseline non-HDL-C levels.

**Conclusions:**

The interval decline in lower-baseline total cholesterol, LDL-C, and non-HDL-C levels was linked to a higher risk for all-cause cancer incidence. More attention to a potential cancer risk may be warranted for an unexplained fall in serum lipids.

**Supplementary Information:**

The online version contains supplementary material available at 10.1186/s12944-021-01570-1.

## Background

Cancer has been the leading cause of death in Taiwan for several decades [1], and its incidence has been increasing. The number of cancer patients by age-standardized incidence rate increased from 191.6 per 100,000 people in 1996 to 309.8 in 2018 [2]. The well-known risk factors for malignant neoplasm included older age, family history, certain types of infection, and substance exposure such as alcohol or tobacco [3]. Furthermore, blood lipid metabolic dysregulation has also been correlated with increased carcinogenic risk [4]. Lipids are fundamental components in cellular homeostasis as they provide energy, stabilize the phospholipid bilayer in the plasma membrane, and are involved in various intracellular signal transduction pathways [4]. Dysregulation of lipid metabolism can activate several essential oncogenic signaling networks [5–9].

Different serum lipid components are associated with risk of various types of cancer. For example, higher total cholesterol (TC) levels are associated with higher risk of prostate and colon cancers in men and breast cancer in women [10]. Higher triglyceride levels and lower high-density lipoprotein cholesterol (HDL-C) levels are associated with breast and lung cancer risk [11, 12]. However, conflicting results in various studies suggest no relationship or even an inverse association between plasma lipids and cancer development [4, 13–17].

The high metabolic rate of cancer cell proliferation may have an impact on the serum lipid levels [4]. Earlier studies have demonstrated a decline in serum cholesterol levels during cancer development [18, 19]. Therefore, the effects of preclinical cancer could demonstrate normal or even lower serum cholesterol values among pre-existing cancer patients [14]. Most studies either compared baseline serum lipids with cancer risk or followed the study population for a relatively short duration. Hence, the effect of preclinical cancer could not be clarified. In addition, the different trajectories of serum lipid changes may differentially influence cell metabolism. Thus, studies focusing on the interval change of lipids before cancer diagnosis are of great value to elucidate the exact correlation between serum lipid and cancer risk. This prospective cohort study aimed to investigate the association of baseline and changes in various serum lipid levels on the risk of cancer incidence.

## Methods

### Study population and data source

**Fig. 1:**
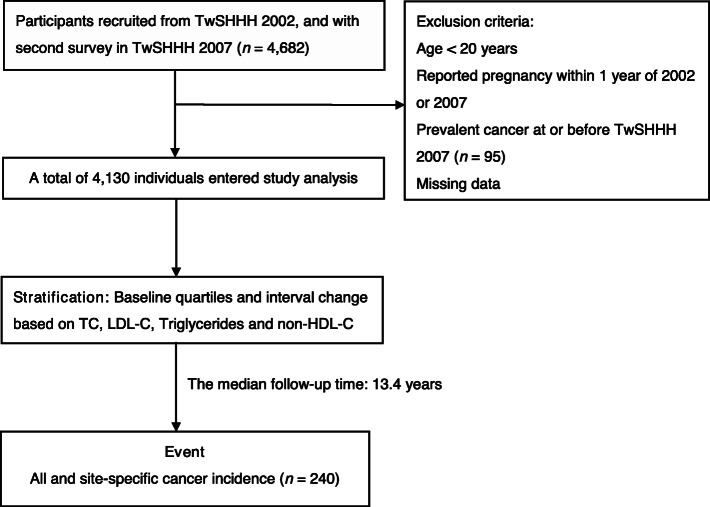
Flow chart of the participants in the study cohort. TwSHHH, Taiwanese Survey on Prevalence of Hypertension, Hyperglycemia, and Hyperlipidemia; TC, total cholesterol; LDL-C, low density lipoprotein cholesterol; non-HDL-C, non-high-density lipoprotein cholesterol. Model adjusted for age, sex, body mass index, current smoking, alcohol drinking, betel nuts consumption, regular exercise, marital status, education level, income level, diabetes mellitus, hypertension, high-sensitivity C-reactive protein, menopause status, hormone replacement therapy and lipid-lowering agent use. Incidence rate is shown per 1000 person-years. TC, total cholesterol; LDL-C, low density lipoprotein cholesterol; BMI, body mass index

This is a population-based prospective cohort study. Participants were identified using population registries maintained in the Taiwanese Survey on Prevalence of Hypertension, Hyperglycemia, and Hyperlipidemia (TwSHHH) in 2002. TwSHHH 2002 was a general health survey launched for national population cohorts, and proposed a standard protocol for data collection. The follow-up evaluation was carried out from June 2007 to May 2008 in TwSHHH 2007. The dataset was also linked to Taiwan’s National Health Insurance Research Database (NHIRD). The NHIRD offered access to outpatient visits, hospitalization records, prescribed medications, and National Death Registry. The flow chart of the study design is shown in Fig. 1.

This study excluded participants who were younger than 20 years, had prevalent cancer at or prior to the enrolment in TwSHHH 2007, reported pregnancy within 1 year prior to TwSHHH 2002 or TwSHHH 2007, and missing data. The final cohort population consisted of 4130 participants (2185 women and 1945 men). This study was in adherence with the Declaration of Helsinki. The protocol was reviewed and approved by the Research Ethics Committee of National Taiwan University Hospital. The committee complied with the Good Clinical Practice Guidelines (NTUH-REC number: 201901103 W [Institutional Review Board reference]).

### Data collection

In TwSHHH 2002, all the participants responded to a standardized self-administered questionnaire and received routine examinations. The questionnaire included socio-demographic characteristics such as smoking status, alcoholic drinking, betel nuts consumption, exercise habits, menopause status, and medical and family history. Women participants were additionally interviewed with hormone replacement therapy use. Hypertension was diagnosed when systolic blood pressure ≥ 140 mmHg or diastolic blood pressure ≥ 90 mmHg in consecutive measurements, or when on anti-hypertensive agents [20]. Diabetes mellitus was diagnosed with a fasting blood sugar level of ≥126 mg/dL and hemoglobin A1c level ≥ 6.5 mg/dL, or when on antidiabetic medication [21].

Blood samples were drawn after a 12 h of overnight fasting. Low-density lipoprotein cholesterol (LDL-C) was estimated using the Friedewald formula of “TC - HDL-C - (triglycerides/5)” in TwSHHH 2002 and was measured directly by homogeneous assays in TwSHHH 2007 [22]. TC and triglycerides were obtained using colorimetry [23]. HDL-C was obtained using electrophoresis. Non-HDL cholesterol (Non-HDL-C) was calculated as subtracting HDL-C from TC.

For the baseline analysis, participants were divided into quartiles according to the levels of each lipid component in TwSHHH 2002. For the interval changes in lipid levels between TwSHHH 2002 and 2007, participants were divided into six categories (low-decreased, low-stable, low-increased, high-decreased, high-stable, and high-increased). Low groups consisted of participants with baseline lipid levels categorized into quartiles 1 and 2, while high groups consisted of those with baseline lipid levels categorized into quartiles 3 and 4. The stable groups were defined as those with a lipid change of less than 0.25 standard deviation in baseline values of serum lipids. The increased groups were defined as those with a positive lipid change of greater than or equal to 0.25 standard deviation in baseline values of serum lipids. The decreased groups were defined as those with a negative lipid change of greater than or equal to 0.25 standard deviation in baseline values of serum lipids. Low-decreased group was defined as the reference group based on previous evidence that lower baseline or declining serum lipid level was associated with cancer development [18, 19].

### Outcome identification and follow-up

The follow-up for each participant started since the index date in which blood samples were obtained in TwSHHH 2002 and ended on the date of cancer diagnosis, death, loss of follow-up, or administrative censoring (December 31, 2015). The outcomes were identified in accordance with the International Classification of Diseases, Ninth Revision, Clinical Modification (ICD9-CM) codes. Both outpatient and inpatient records were used to confirm the diagnoses of outcomes. Cancer cases were identified with one or more diagnoses of ICD9-CM codes for malignant neoplasms, including 140–208 from the NHIRD registry database.

### Statistical analyses

Analysis of variance for continuous variables and Chi-squared test for categorical variables were done to assess the differences among groups. The incidence rates of cancer were presented by the event numbers per 1000 person-years of follow-up. Log-rank test was used to compare the cancer incidence rates among different groups of lipid profiles. The multivariable Cox regression models were used to evaluate the relationship of lipid levels with cancer risks. The proportionality assumption was tested and verified [24]. Subgroup analyses were conducted to assess effect modification by age, sex, and body mass index (BMI). Sensitivity analyses were performed to determine the robustness of study findings: (1) excluding cancer cases or death in 1 year from index date of TwSHHH 2007, (2) excluding participants who used lipid-lowering agents, and (3) excluding extremely high triglyceride levels (> 400 mg/dL). An additional analysis was carried out to explore the correlation between the changes in body weight and blood lipids. A two-tailed *P*-value of < 0.05 was considered statistically significant differences. SAS version 9.4 (SAS Institute, Cary, North Carolina, U.S.) and Stata version 14 (Stata Corporation, College Station, Texas, USA) were used as analytics tools.

## Results

### Study characteristics

Table 1 shows the demographic characteristics of the study cohort categorized according to baseline serum LDL-C quartiles. A total of 4130 participants were included in this study. A total of 2,004,174 person-years at risk was recognized with a median follow-up period of 13.4 years. The mean age at study entry was 44.9 years. A higher LDL-C quartile was positively associated with age, BMI, regular exercise, married status, diabetes mellitus, hypertension, high-sensitivity C-reactive protein levels, menopause, use of lipid-lowering agents, and inversely associated with female gender and educational level. During follow-up, a total of 240 cancer events were ascertained.

**Table 1:** Distribution of demographic characteristics stratified by quartiles of baseline LDL-C in the cohort population (*N* = 4130)

Variable	Total	Quartiles (mg/dL)				*P* value
		Q1 (38–96)	Q2 (97–113)	Q3 (114–132)	Q4 (133–252)	
Women (n, %)	2185 (52.9)	537 (58.8)	570 (53.8)	547 (50.1)	531 (49.9)	<.001
Age (years, %)						<.001
20–64	3590 (87.0)	848 (92.9)	947 (89.4)	924 (84.6)	871 (81.9)	
≥ 65	538 (13.0)	65 (7.1)	112 (10.6)	168 (15.4)	193 (18.1)	
BMI (kg/m^2^, %)						<.001
< 18.5	202 (5.7)	81 (10.3)	62 (6.8)	39 (4.2)	20 (2.2)	
18.5–23.9	1916 (53.7)	497 (63.2)	531 (58.0)	483 (51.5)	405 (43.7)	
24–26.9	917 (25.7)	137 (17.4)	219 (23.9)	264 (28.1)	297 (32.0)	
≥ 27	532 (14.9)	71 (9.0)	104 (11.4)	152 (16.2)	205 (22.1)	
Current smoker (n, %)	822 (19.9)	170 (18.6)	219 (20.7)	227 (20.8)	206 (19.4)	0.56
Alcohol drinking (n, %)	985 (23.9)	212 (23.2)	269 (25.4)	260 (23.8)	244 (22.9)	0.55
Betel nuts consumption (n, %)	298 (7.2)	72 (7.9)	84 (7.9)	74 (6.8)	68 (6.4)	0.42
Regular exercise (n, %)	962 (23.3)	204 (22.3)	218 (20.6)	287 (26.3)	253 (23.8)	0.015
Married (n, %)	2569 (62.2)	482 (52.8)	619 (58.5)	737 (67.5)	731 (68.7)	<.001
Education level						<.001
Lower education (n, %)	2143 (51.9)	403 (44.1)	495 (46.7)	590 (54.0)	655 (61.6)	
Higher education (n, %)	1985 (48.1)	510 (55.9)	564 (53.3)	502 (46.0)	409 (38.4)	
Low income level (n, %)	2940 (71.2)	674 (73.8)	739 (69.8)	760 (69.6)	767 (72.1)	0.12
Hypertension (n, %)	237 (5.7)	29 (3.2)	39 (3.7)	73 (6.7)	96 (9.0)	<.001
Diabetes mellitus (n, %)	192 (4.7)	26 (2.9)	31 (2.9)	58 (5.3)	77 (7.2)	<.001
hsCRP (mg/L)	0.24 (0.55)	0.17 (0.39)	0.21 (0.47)	0.27 (0.75)	0.27 (0.48)	<.001
Menopause (n, %)	771 (18.7)	85 (9.3)	157 (14.8)	227 (20.8)	302 (28.4)	<.001
Hormone replacement therapy (n, %)	357 (8.7)	80 (8.8)	87 (8.2)	94 (8.6)	96 (9.0)	0.93
Lipid-lowering agent use (n, %)	624 (15.1)	49 (5.4)	92 (8.7)	172 (15.8)	311 (29.2)	<.001
Lipid profile (mg/dL)						
TC (*n* = 4128)		146.0 (20.1)	171.7 (21.4)	192.3 (16.6)	230.0 (29.8)	<.001
LDL-C (*n* = 4128)		84.6 (9.1)	105.0 (4.8)	122.3 (5.3)	153.1 (18.8)	<.001
Triglycerides (*n* = 4099)		94.6 (69.0)	119.9 (83.3)	137.8 (82.1)	164.2 (92.5)	<.001
Non-HDL-C (*n* = 4128)		94.3 (19.3)	116.7 (21.0)	136.2 (15.1)	171.5 (26.7)	<.001
∆TC (*n* = 3842)		8.7 (28.7)	1.0 (26.9)	−2.0 (29.4)	−21.9 (39.6)	<.001
∆LDL-C (*n* = 3842)		3.8 (24.2)	−0.7 (24.2)	−2.8 (28.0)	−18.3 (34.2)	<.001
∆Triglycerides (*n* = 3817)		2.8 (64.7)	−2.2 (75.3)	−1.2 (84.0)	−10.5 (86.1)	0.002
∆Non-HDL-C (*n* = 3842)		7.7 (26.3)	4.0 (25.3)	2.3 (29.0)	−15 (37.6)	<.001

*LDL-C* Low density lipoprotein cholesterol, *BMI* Body mass index, *hsCRP* High-sensitivity C-reactive protein, *TC* Total cholesterol, *Non-HDL-C* Non-high-density lipoprotein cholesterol; ∆, interval change between the Taiwanese Survey on Prevalence of Hypertension, Hyperglycemia, and Hyperlipidemia in 2002 and follow-up measurement in 2007

### Relationship between baseline and change of lipid levels and all-cancer incidence

**Fig. 2:**
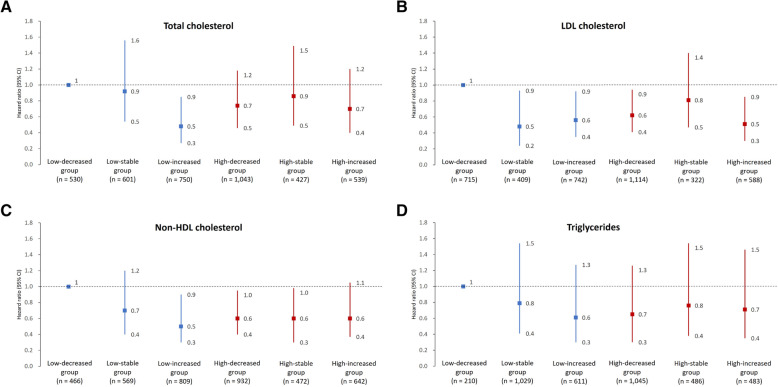
The effects of interval changes in various lipid components for all-cause cancer risk during median 13.4-year follow-up. Model adjusted for age, sex, body mass index, current smoking, alcohol drinking, betel nut consumption, regular exercise, marital status, education level, income level, diabetes mellitus, hypertension, high-sensitivity C-reactive protein, menopause status, hormone replacement therapy, and lipid-lowering agent use. Incidence rate is shown per 1000 person-years. CI, confidence interval; LDL, low density lipoprotein; HDL, high density lipoprotein

The baseline lipid quartiles were not associated with total cancer incidence across all categories of lipid components (Table 2). Figure 2 demonstrates the relative risks of all-cause cancer incidence based on the interval changes of each lipid component. Participants in low-increased group of TC component exhibited a lower risk for all-cause cancer incidence compared to the low-decreased group (adjusted hazard ratio [aHR], 0.48; 95% confidence interval [CI], 0.27 to 0.85). For LDL-C component, compared to participants in the low-decreased group, those in the low-stable (aHR, 0.48; 95% CI, 0.24 to 0.93), low-increased (aHR, 0.56; 95% CI, 0.35 to 0.92), high-decreased (HR, 0.62; 95% CI, 0.41 to 0.94), and high-increased groups (aHR, 0.51; 95% CI, 0.30 to 0.85) showed lower risk of all-cause cancer incidence. After combining all other groups as a reference group, the low-decreased group for the LDL-C component also revealed a higher cancer risk with the adjusted hazard ratio of 1.59 (95% CI, 1.00–2.55) (Table S1 in Additional file 1). For non-HDL-C component, the results yielded a lower risk of all-cause cancer incidence for participants in the low-increased (aHR, 0.54; 95% CI, 0.31 to 0.92), high-decreased (aHR, 0.58; 95% CI, 0.35 to 0.95), and high-stable groups (aHR, 0.55; 95% CI, 0.30 to 0.98) in comparison to participants in the low-decreased group.

**Table 2:** The adjusted relative risks and 95% confidence intervals of all-cause cancer incidence according to quartiles of each lipid component

Variable	Q1	Q2	Quartiles				*P* value
			*P* value	Q3	*P* value	Q4	
TC							
Range, mg/dL	82–158	159–180		181–206		207–660	
Cases	46	57		66		71	
Incidence rate	3.7	4.1		4.8		4.9	
Model 1	1	1.01 (0.65–1.57)	0.97	1.25 (0.82–1.90)	0.30	1.07 (0.70–1.63)	0.76
Model 2	1	0.95 (0.61–1.47)	0.81	1.09 (0.72–1.66)	0.69	0.91 (0.59–1.39)	0.65
Model 3	1	1.00 (0.63–1.58)	0.99	1.07 (0.69–1.67)	0.77	0.92 (0.58–1.46)	0.73
LDL-C							
Range, mg/dL	38–96	97–113		114–132		133–252	
Cases	43	61		71		65	
Incidence rate	3.5	4.3		4.9		4.6	
Model 1	1	1.12 (0.73–1.73)	0.61	1.18 (0.77–1.80)	0.45	1.01 (0.66–1.56)	0.96
Model 2	1	1.04 (0.67–1.61)	0.85	1.02 (0.66–1.56)	0.94	0.86 (0.55–1.33)	0.50
Model 3	1	1.07 (0.68–1.69)	0.76	0.93 (0.59–1.48)	0.77	0.89 (0.56–1.42)	0.63
TG							
Range, mg/dL	28–74	75–102		103–152		153–774	
Cases	46	55		62		76	
Incidence rate	3.6	4.2		4.4		5.4	
Model 1	1	1.01 (0.66–1.56)	0.95	1.00 (0.66–1.53)	0.98	1.06 (0.70–1.61)	0.79
Model 2	1	0.89 (0.57–1.38)	0.60	0.83 (0.54–1.27)	0.38	0.82 (0.53–1.28)	0.38
Model 3	1	0.80 (0.50–1.27)	0.34	0.80 (0.51–1.25)	0.33	0.82 (0.52–1.31)	0.41
Non-HDL-C							
Range, mg/dL	55–104	105–124		125–148		149–631	
Cases	43	60		66		71	
Incidence rate	3.5	4.4		4.5		5.0	
Model 1	1	1.03 (0.66–1.59)	0.90	1.06 (0.69–1.61)	0.80	0.99 (0.65–1.52)	0.97
Model 2	1	0.92 (0.59–1.43)	0.71	0.87 (0.56–1.33)	0.52	0.80 (0.51–1.23)	0.31
Model 3	1	0.92 (0.58–1.45)	0.70	0.79 (0.50–1.26)	0.32	0.82 (0.51–1.31)	0.40

Model 1: adjusted for age and sex

Model 2: Model 1 and additionally adjusted for body mass index, current smoking, alcohol drinking, betel nuts consumption, regular exercise, marital status, education level and income level

Model 3: Model 2 and additionally adjusted for diabetes mellitus, hypertension, high-sensitivity C-reactive protein, menopause status, hormone replacement therapy and lipid-lowering agent use

Incidence rate is shown per 1000 person-years, *TC* Total cholesterol, *LDL-C* Low density lipoprotein cholesterol, *Non-HDL-C* Non-high-density lipoprotein cholesterol, *TG* Triglycerides

### Subgroup and sensitivity analyses

The relative risks of all-cause cancer incidence based on the baseline quartiles and interval changes in TC and LDL-C components stratified by different variables are shown in Table S2 (Additional file 2) and Table 3, respectively. The results did not substantially vary when stratified by age, gender, or BMI for all categories of baseline lipid components. The effects of interval changes on the lipid levels were consistent across subgroups. The results of sensitivity analysis remained robust by excluding cancer events or death in 1 year from index date of TwSHHH 2007, excluding the participants using lipid-lowering agents and cases with extremely high triglyceride levels (> 400 mg/dL; Table S3 in Additional file 3). The additional analysis showed that the body weight of most participants remained constant or slightly increased between the follow-up intervals (Tables S4–1 and S4–2 in Additional file 4). The overall estimates also remained mostly stable for the TC, LDL-C, and Non-HDL-C components after excluding individuals with interval body weight loss (Table S4–3 in Additional file 4).

**Table 3:** Subgroup analysis for the effects of interval changes in TC and LDL-C components for all-cause cancer risk during median 13.4-year follow-up

Variable	Low-decreased	Low-stable	Low-increased	High-decreased	High-stable	High-increased	***P*** value for interaction
**TC**							
Gender							
women	1	1.07 (0.46–2.50)	0.60 (0.25–1.43)	0.74 (0.34–1.59)	0.79 (0.32–1.95)	0.66 (0.28–1.58)	0.92
men	1	0.85 (0.43–1.69)	0.39 (0.18–0.88)	0.65 (0.36–1.18)	0.85 (0.41–1.76)	0.73 (0.35–1.48)	
Age							
< 65 years old	1	0.86 (0.46–1.59)	0.36 (0.17–0.74)	0.80 (0.46–1.42)	0.74 (0.37–1.47)	0.69 (0.37–1.30)	0.33
≥ 65 years old	1	1.08 (0.36–3.27)	0.84 (0.32–2.26)	0.60 (0.25–1.41)	1.24 (0.45–3.41)	0.75 (0.24–2.34)	
BMI							
< 24 kg/m^2^	1	1.33 (0.75–2.36)	0.61 (0.32–1.14)	0.95 (0.55–1.63)	0.87 (0.44–1.73)	0.70 (0.36–1.37)	0.80
≥ 24 kg/m^2^	1	0.62 (0.27–1.40)	0.32 (0.12–0.84)	0.60 (0.32–1.14)	0.63 (0.29–1.37)	0.56 (0.26–1.21)	
**LDL-C**							
Gender							
women	1	0.24 (0.08–0.70)	0.42 (0.20–0.85)	0.37 (0.20–0.71)	0.76 (0.37–1.60)	0.29 (0.12–0.66)	0.11
men	1	0.84 (0.35–2.04)	0.76 (0.38–1.52)	0.86 (0.49–1.53)	0.78 (0.33–1.82)	0.76 (0.38–1.51)	
Age							
< 65 years old	1	0.42 (0.19–0.91)	0.51 (0.29–0.89)	0.59 (0.36–0.98)	0.81 (0.42–1.54)	0.45 (0.24–0.82)	0.98
≥ 65 years old	1	0.74 (0.19–2.81)	0.73 (0.26–2.08)	0.69 (0.31–1.53)	1.02 (0.35–2.92)	0.64 (0.24–1.73)	
BMI							
< 24 kg/m^2^	1	0.55 (0.27–1.12)	0.65 (0.38–1.10)	0.63 (0.39–1.02)	0.76 (0.40–1.44)	0.54 (0.29–0.98)	0.99
≥ 24 kg/m^2^	1	0.44 (0.15–1.32)	0.51 (0.23–1.12)	0.61 (0.33–1.11)	0.73 (0.33–1.62)	0.48 (0.23–1.01)	

## Discussion

This prospective cohort study of initially healthy Taiwanese adults investigated the association of various lipid biomarkers and their interval changes with risk of all-cause cancer incidence during the median follow-up time of 13.4 years. The baseline serum lipid levels did not associate with incidence of total cancer. Participants with constantly stable or positively-changed cholesterol levels showed a lower cancer risk compared with those with initially low and subsequently decreased lipid levels. The association was mainly suggested from the components of TC, LDL-C, and non-HDL-C.

The evidence regarding the relationship between serum lipids and risk of cancer has been inconsistent. Several studies observed an inverse association, especially during first few years from the study onset [15, 17, 25–28]. The preclinical effect of cancer consuming more cholesterol on tumor growth could introduce potential reverse causality. However, some studies found the inverse association persisted even more than 10 years before the diagnosis of cancer, which could not be entirely attributed to the preclinical effect [10, 27].

Limited studies have explored the exact trajectories of serum lipid changes before the development of cancer. A Mendelian randomization study showed that lifelong low plasma LDL-C levels caused by gene polymorphisms are unrelated to increased cancer risk [29]. Similarly, this study found that the risk of cancer incidence was not associated with baseline lipid levels, but with their interval changes. Kritchevsky et al. in a cohort study with 103 middle-aged men found a decline in serum lipids approximately 2 years preceding cancer diagnosis, with LDL-C predominantly reflecting the decrease among lipoprotein fractions [18]. Winawer et al. in a case-control study with a modest sample size demonstrated an average decline of 13% in serum cholesterol occurring gradually during the 10 years prior to the diagnosis of colon cancer [19]. In this study, the depletion of serum lipids occurred more than 5 years before malignancy development in almost all cancer events. The median time for the diagnosis of cancer is around 7.7–10.6 years after the decline of serum lipids for various lipid components (Table S5 in Additional file 5). In addition, the exclusion of 1-year incident cases after index date of TwSHHH 2007 did not alter the findings. The results are in line with those of previous studies, that is, the preclinical effect does not entirely contribute to the inverse relationship between lipid change and cancer development.

In this study, the association between the decline in blood lipid levels and cancer risk did not change substantially after exclusion of lipid-lowering agent users. The mainstay of these agents, like statin, is believed to inhibit key enzymes in the cholesterol synthesis pathway and may disrupt oncogenesis [30]. Ambiguous results were obtained with respect to the relationship between the use of lipid-lowering agents and cancer risk. One meta-analysis concluded that reductions in LDL-C with statin treatment did not increase the cancer incidence during a median follow-up time of 5 years [31]. Another study indicated no difference in risk of colorectal cancer between statin continuers and discontinuers [32]. One Mendelian randomization study proposed that statins utilized a cholesterol-independent pathway to reduce the risk of malignancies [33]. In contrast, the association of decreasing blood lipids with cancer risk remained mostly consistent despite medical treatment in this study. Therefore, there may be an independent influence of endogenous metabolic depression on the processes of tumorigenesis and further research is warranted.

In this study, a higher risk of cancer incidence by depletion of serum lipids was mainly drawn from TC, LDL-C, and non-HDL-C components. The effects of TC and non-HDL-C on risk for cancer incidence appeared to be largely affected but could not be fully explained by LDL-C. The potential influence of remnant lipoprotein fractions or their binding apolipoproteins within the non-HDL-C component could not be concluded in the current study. Furthermore, the influence of LDL-C on cancer development may differ among various site-specific malignancies. While some studies found that low LDL-C levels may increase risk for hematological and esophageal cancers [34, 35], other studies reported marginal or non-significant association between LDL-C levels and the risk of breast cancer [36, 37]. The cholesterol requirement and basic constitution vary in different tissues; thus, the tissue origin of the neoplasm may also lead to discrepant observations [38].

The exact pathophysiology regarding the correlation between LDL-C and cancer development has remained inconclusive. The possibility that the depletion of LDL-C might merely act as a surrogate for body weight loss, which frequently occurs during cancer development, was proved to be marginal in this study. The oxidized LDL-C serves as a marker for lipid peroxidation, which could enhance carcinogenesis [34]. LDL particles transport cholesterol to surrounding tissues mainly through receptor-mediated pathways. An accelerated LDL receptor activity resulting in increased intracellular cholesterol influx also precedes the development of certain types of cancer [39, 40]. The activation of phosphatidylinositol 3-kinase/protein kinase B/mammalian target of rapamycin signaling pathway may mediate the upregulation of intracellular cholesterol levels, which is related to cell growth [38]. Furthermore, prolonged depletion of plasma cholesterol may contribute to tumorigenesis by promoting the activation of nuclear factor-κB [41]. It could also disrupt the homeostatic balance of lipid raft and dysregulate tumor cell growth [30]. Finally, the elimination of cholesterol by altered gut microbiota may also facilitate cancer development [30].

### Comparison with other Asian countries

Cancer epidemiology and metabolic characteristics vary among different races and territories. The high prevalence of gastrointestinal cancer in Asian countries may affect the relationship between blood lipid levels and cancer risk. A Korean population-based cohort study found the correlation between TC and risk of all-cancer incidence differed largely by cancer site [10]. Other two Japanese studies indicated an inverse relationship between TC and cancer incidence [15, 25]. The inverse correlation was observed mainly for liver and stomach cancers. One study from China even showed a V-shaped relationship between LDL-C and cancer risk [42]. However, most previous studies lack information concerning change in the lipids before cancer development, which may partly constitute to the discrepancies. Moreover, while stomach cancer is markedly prevalent in most East Asian countries, liver cancer is relatively common in Taiwan [43, 44]. In this study, gastrointestinal cancer accounted for approximately 30%, which corresponded to the cancer epidemiology in Taiwan during the study period [45]. Unfortunately, the influence of metabolic depression on different site-specific cancers could not be studied in detail.

### Study strength and limitations

This study has some important strengths. This prospective cohort study is the first to simultaneously evaluate the association between combined basic and interval changes of blood lipids and cancer risk in a population-based large cohort with a long follow-up time. Although the study population was relatively younger and metabolically healthier than the general population in Taiwan, the extrapolation of the results may still be appropriate due to the utilization of population-based representative databases. Second, this study used standardized measurements of variables. National registers also provided detailed information on cancer diagnosis or death. Third, this study collected comprehensive information on potential confounders.

However, it had a few limitations. First, due to the nature of observational design, a solid causal relationship could not be established. The relatively small number of cancer events and missing data may lessen the power of observed association and hinder further analysis for site-specific cancers. However, this study included various lipoprotein subfractions with a long follow-up time. The findings were consistent across various groups and with sensitivity analysis. Second, the Friedewald formula was used to estimate LDL-C level for the TwSHHH 2002 cohort. Nevertheless, the results did not change after exclusion of participants with extremely high triglyceride levels. Third, other residual confounding, such as diet, other medication history (like oral contraceptive or aspirin), and family history were lacking in this study.

## Conclusions

In this population-based prospective cohort study, the interval decline of lower-baseline serum lipid levels was associated with increased risk for all-cause cancer incidence. The effect was mainly suggested by TC, LDL-C, and non-HDL-C components. The results provide novel evidence regarding the impact of plasma lipid dynamics on cancer development. Despite the protective role of lower plasma lipid levels in cardiovascular disease, an unexplained decline in serum lipids may imply a potential risk of cancer development. Therefore, clinicians may pay attention to monitoring and maintaining serum lipids for individuals with unexplained drop in serum lipids. Further research focusing on the effects of lipid change among different site-specific cancers is also warranted.

## Supplementary Information

**Additional file 1: Table S1**: Adjusted relative risks and 95% confidence intervals of all-cause cancer incidence in the low-decreased group against all other groups for the LDL-C component.

**Additional file 2: Table S2**: Subgroup analysis for the adjusted relative risks and 95% confidence intervals of all-cause cancer incidence according to quartiles of TC and LDL-C components.

**Additional file 3: Table S3**: Sensitivity analysis for the adjusted relative risks and 95% confidence intervals of all-cause cancer incidence according to quartiles and interval changes of each lipid component.

**Additional file 4: Table S4–1**: Distribution of BMI values for interval changes in the LDL-C component in the cohort population. **Table S4–2**: BMI change between TwSHHH 2002 and TwSHHH 2007 for the LDL-C component in the cohort population. **Table S4–3**: Sensitivity analysis for the adjusted relative risks and 95% confidence intervals of all-cause cancer incidence according to the interval changes in each lipid component.

**Additional file 5: Table S5**: Time distribution of cancer diagnosis after TwSHHH 2002 according to the interval changes in each lipid component.

## Data Availability

The datasets generated and analyzed during the current study are available from the corresponding author on reasonable request.

## Abbreviations

TC: Total cholesterol
HDL-C: High-density lipoprotein cholesterol
TwSHHH: Taiwanese Survey on Prevalence of Hypertension, Hyperglycemia, and Hyperlipidemia
NHIRD: Taiwan’s National Health Insurance Research Database
LDL-C: Low-density lipoprotein cholesterol
Non-HDL-C: Non-HDL cholesterol
ICD9-CM: International Classification of Diseases, Ninth Revision, Clinical Modification
BMI: Body mass index
aHR: Adjusted hazard ratio
CI: 95% confidence interval
