# Supplementary material for: Lipid biomarkers and Cancer risk - a population-based prospective cohort study in Taiwan
Source: Lipids Health Dis. 2021 Oct 10;20:133. doi: 10.1186/s12944-021-01570-1 (PMC8502377; doi:10.1186/s12944-021-01570-1)
Supplement: Supplementary file 1 — Additional file 1: Table S1: Adjusted relative risks and 95% confidence intervals of all-cause cancer incidence in the low-decreased group against all other groups for the LDL-C component. [file 12944_2021_1570_MOESM1_ESM.doc]

**Table S1.** **Adjusted relative risks and 95% confidence intervals of all-cause cancer incidence in the low-decreased group against all other groups for the LDL-C component**

| **Variable** | **All other groups**† | **Low-decreased** |
| --- | --- | --- |
| Model 1 | 1 | 1.38 (0.87–2.22) |
| Model 2 | 1 | 1.58 (0.99–2.52) |
| Model 3 | 1 | 1.59 (1.00–2.55) |

†Combining low-stable, low-increased, high-decreased, high-stable, and high-increased groups as a reference group

Model 1: adjusted for age and sex.

Model 2: Model 1 and additionally adjusted for body mass index, current smoking, alcohol drinking, betel nut consumption, regular exercise, marital status, education level, and income level.

Model 3: Model 2 and additionally adjusted for diabetes mellitus, hypertension, high-sensitivity C-reactive protein, menopause status, hormone replacement therapy, and lipid-lowering agent use.

LDL-C, low density lipoprotein cholesterol.
