# Supplementary material for: Lipid biomarkers and Cancer risk - a population-based prospective cohort study in Taiwan
Source: Lipids Health Dis. 2021 Oct 10;20:133. doi: 10.1186/s12944-021-01570-1 (PMC8502377; doi:10.1186/s12944-021-01570-1)
Supplement: Supplementary file 2 — Additional file 2: Table S2: Subgroup analysis for the adjusted relative risks and 95% confidence intervals of all-cause cancer incidence according to quartiles of TC and LDL-C components. [file 12944_2021_1570_MOESM2_ESM.doc]

| **Table S2. Subgroup analysis for the adjusted relative risks and 95% confidence intervals of all-cause cancer incidence according to quartiles of TC and LDL-C components** | **Quartiles** | | | |  | |
| --- | --- | --- | --- | --- | --- | --- |
| **Variable** | Q1 | Q2 | Q3 | Q4 | | ***P* value for interaction** |
| **TC** |  |  |  |  | |  |
| Gender |  |  |  |  | |  |
| women | 1 | 0.92 (0.46-1.84) | 0.89 (0.45-1.76) | 0.80 (0.40-1.59) | | 0.95 |
| men | 1 | 0.99 (0.54-1.83) | 1.08 (0.60-1.95) | 0.86 (0.47-1.61) | |  |
| Age |  |  |  |  | |  |
| < 65 years old | 1 | 1.26 (0.72-2.18) | 1.33 (0.77-2.30) | 1.24 (0.71-2.17) | | 0.10 |
| ≥ 65 years old | 1 | 0.56 (0.24-1.29) | 0.65 (0.30-1.40) | 0.46 (0.21-1.03) | |  |
| BMI |  |  |  |  | |  |
| < 24 kg/m^2^ | 1 | 1.25 (0.69-2.27) | 1.27 (0.70-2.33) | 0.86 (0.44-1.70) | | 0.46 |
| ≥ 24 kg/m^2^ | 1 | 1.25 (0.68-2.27) | 1.28 (0.70-2.34) | 0.85 (0.43-1.67) | |  |
| **LDL-C** |  |  |  |  | |  |
| Gender |  |  |  |  | |  |
| women | 1 | 1.37 (0.70-2.66) | 0.86 (0.41-1.79) | 0.92 (0.45-1.90) | | 0.34 |
| men | 1 | 0.86 (0.46-1.61) | 0.96 (0.53-1.74) | 0.77 (0.41-1.42) | |  |
| Age |  |  |  |  | |  |
| < 65 years old | 1 | 1.34 (0.79-2.28) | 1.02 (0.59-1.79) | 1.10 (0.63-1.92) | | 0.13 |
| ≥ 65 years old | 1 | 0.59 (0.23-1.51) | 0.78 (0.34-1.78) | 0.51 (0.22-1.18) | |  |
| BMI |  |  |  |  | |  |
| < 24 kg/m^2^ | 1 | 1.32 (0.74-2.36) | 1.22 (0.67-2.22) | 0.65 (0.32-1.31) | | 0.06 |
| ≥ 24 kg/m^2^ | 1 | 1.32 (0.74-2.36) | 1.22 (0.67-2.21) | 0.64 (0.32-1.30) | |  |

Model adjusted for age, sex, body mass index, current smoking, alcohol drinking, betel nuts consumption, regular exercise, marital status, education level, income level, diabetes mellitus, hypertension, high-sensitivity C-reactive protein, menopause status, hormone replacement therapy and lipid-lowering agent use. Incidence rate is shown per 1,000 person-years. TC, total cholesterol; LDL-C, low density lipoprotein cholesterol; BMI, body mass index.

†TG > 400 mg/dL. TC, total cholesterol; LDL-C, low density lipoprotein cholesterol; TG, triglycerides; HDL-C, high density lipoprotein cholesterol.
