# Supplementary material for: Lipid biomarkers and Cancer risk - a population-based prospective cohort study in Taiwan
Source: Lipids Health Dis. 2021 Oct 10;20:133. doi: 10.1186/s12944-021-01570-1 (PMC8502377; doi:10.1186/s12944-021-01570-1)
Supplement: Supplementary file 3 — Additional file 3: Table S3: Sensitivity analysis for the adjusted relative risks and 95% confidence intervals of all-cause cancer incidence according to quartiles and interval changes of each lipid component. [file 12944_2021_1570_MOESM3_ESM.doc]

**Table ~~S2~~S3. Sensitivity analysis for the adjusted relative risks and 95% confidence intervals of all-cause cancer incidence according to quartiles and interval changes of each lipid component**

†TG > 400 mg/dL. TC, total cholesterol; LDL-C, low density lipoprotein cholesterol; TG, triglycerides; Non-HDL-C, ~~N~~non-high-density lipoprotein cholesterol; Model adjusted for age, sex, body mass index, current smoking, alcohol drinking, betel nut consumption, regular exercise, marital status, education level, income level, diabetes mellitus, hypertension, high-sensitivity C-reactive protein, menopause status, hormone replacement therapy, and lipid-lowering agent use.

|  | **Quartiles** | | | | | | | |
| --- | --- | --- | --- | --- | --- | --- | --- | --- |
|  | Q1 | | Q2 | | Q3 | | Q4 | |
| **Exclusion of the first 1-year incident cases** |  | |  | |  | |  | |
| TC | 1 | | 0.98 (0.61-1.57) | | 1.05 (0.66-1.66) | | 0.89 (0.56-1.44) | |
| LDL-C | 1 | | 1.07 (0.67-1.70) | | 0.85 (0.53-1.38) | | 0.90 (0.55-1.45) | |
| TG | 1 | | 0.88 (0.54-1.45) | | 0.93 (0.58-1.50) | | 0.91 (0.55-1.50) | |
| Non-HDL-C | 1 | | 0.90 (0.56-1.45) | | 0.74 (0.45-1.19) | | 0.81 (0.50-1.30) | |
| **Exclusion of cases with extremely high TG level^†^** |  | |  | |  | |  | |
| TC | 1 | | 0.99 (0.63-1.56) | | 1.01 (0.64-1.58) | | 0.85 (0.53-1.36) | |
| LDL-C | 1 | | 1.02 (0.64-1.61) | | 0.84 (0.53-1.34) | | 0.87 (0.54-1.39) | |
| TG | 1 | | 0.81 (0.51-1.28) | | 0.80 (0.51-1.26) | | 0.77 (0.47-1.24) | |
| Non-HDL-C | 1 | | 0.92 (0.58-1.45) | | 0.81 (0.51-1.28) | | 0.74 (0.46-1.19) | |
|  | Low-decreased | Low-stable | | Low-increased | High-decreased | High-stable | | High-increased |
| **Exclusion of the first 1-year incident cases** |  |  | |  |  |  | |  |
| TC | 1 | 0.95 (0.55-1.66) | | 0.50 (0.27-0.91) | 0.74 (0.45-1.21) | 0.89 (0.50-1.60) | | 0.70 (0.40-1.25) |
| LDL-C | 1 | 0.47 (0.23-0.94) | | 0.58 (0.35-0.96) | 0.63 (0.41-0.97) | 0.82 (0.47-1.45) | | 0.42 (0.24-0.74) |
| TG | 1 | 0.69 (0.35-1.35) | | 0.53 (0.25-1.13) | 0.62 (0.32-1.20) | 0.73 (0.36-1.49) | | 0.68 (0.33-1.40) |
| Non-HDL-C | 1 | 0.68 (0.38-1.21) | | 0.55 (0.31-0.96) | 0.56 (0.34-0.95) | 0.55 (0.30-1.01) | | 0.58 (0.33-1.01) |
| **Exclusion of cases with extremely high TG level^†^** |  |  | |  |  |  | |  |
| TC | 1 | 0.93 (0.55-1.58) | | 0.48 (0.27-0.85) | 0.70 (0.43-1.12) | 0.80 (0.45-1.42) | | 0.65 (0.37-1.14) |
| LDL-C | 1 | 0.49 (0.25-0.96) | | 0.59 (0.36-0.97) | 0.60 (0.39-0.92) | 0.81 (0.46-1.42) | | 0.53 (0.32-0.89) |
| TG | 1 | 0.78 (0.40-1.52) | | 0.61 (0.30-1.27) | 0.64 (0.33-1.24) | 0.74 (0.37-1.51) | | 0.65 (0.31-1.35) |
| Non-HDL-C | 1 | 0.70 (0.40-1.21) | | 0.54 (0.31-0.93) | 0.57 (0.34-0.94) | 0.51 (0.28-0.94) | | 0.60 (0.35-1.02) |
| **Exclusion of cases under lipid-lowering agents** |  |  | |  |  |  | |  |
| TC | 1 | 0.90 (0.53-1.54) | | 0.47 (0.26-0.84) | 0.66 (0.41-1.07) | 0.84 (0.47-1.50) | | 0.67 (0.38-1.18) |
| LDL-C | 1 | 0.49 (0.25-0.96) | | 0.56 (0.34-0.93) | 0.61 (0.39-0.94) | 0.79 (0.44-1.41) | | 0.49 (0.28-0.84) |
| TG | 1 | 0.75 (0.38-1.46) | | 0.58 (0.28-1.20) | 0.58 (0.30-1.14) | 0.75 (0.37-1.52) | | 0.59 (0.28-1.27) |
| Non-HDL-C | 1 | 0.73 (0.41-1.28) | | 0.55 (0.31-0.96) | 0.56 (0.33-0.94) | 0.63 (0.35-1.15) | | 0.60 (0.35-1.05) |
