# Supplementary material for: Lipid biomarkers and Cancer risk - a population-based prospective cohort study in Taiwan
Source: Lipids Health Dis. 2021 Oct 10;20:133. doi: 10.1186/s12944-021-01570-1 (PMC8502377; doi:10.1186/s12944-021-01570-1)
Supplement: Supplementary file 4 — Additional file 4: Table S4–1: Distribution of BMI values for interval changes in the LDL-C component in the cohort population. Table S4–2: BMI change between TwSHHH 2002 and TwSHHH 2007 for the LDL-C component in the cohort population. Table S4–3: Sensitivity analysis for the adjusted relative risks and 95% confidence intervals of all-cause cancer incidence according to the interval changes in each lipid component. [file 12944_2021_1570_MOESM4_ESM.doc]

| **Variable** | **Low-decreased** | | **Low-stable** | | **Low-increased** | | **High-decreased** | | **High-stable** | | **High-increased** | |
| --- | --- | --- | --- | --- | --- | --- | --- | --- | --- | --- | --- | --- |
|  | TwSHHH  2002 | TwSHHH  2007 | TwSHHH  2002 | TwSHHH  2007 | TwSHHH  2002 | TwSHHH  2007 | TwSHHH  2002 | TwSHHH  2007 | TwSHHH  2002 | TwSHHH  2007 | TwSHHH  2002 | TwSHHH  2007 |
| BMI (kg/m^2^) | 22.3 (3.7) | 22.4 (3.9) | 22.4 (3.4) | 22.6 (3.5) | 23.2 (3.4) | 23.4 (3.6) | 24.2 (3.6) | 24.5 (3.6) | 24.1 (3.7) | 24.3 (3.8) | 24.3 (3.5) | 24.5 (3.7) |
| BMI (kg/m^2^, %) |  |  |  |  |  |  |  |  |  |  |  |  |
| <18.5 | 40 (12.1) | 35 (10.4) | 75 (8.7) | 76 (8.6)) | 19 (5.1) | 18 (4.6) | 31 (4.8) | 30 (4.4) | 16 (2.2) | 16 (2.1)) | 7 (2.4) | 7 (2.3) |
| 18.5-23.9 | 206 (62.4) | 211 (62.6) | 541 (62.9) | 545 (61.4) | 215 (57.3) | 217 (55.8) | 289 (44.7) | 275 (40.1) | 375 (50.9) | 379 (49.0) | 148 (50.3) | 149 (47.9) |
| 24-26.9 | 53 (16.1) | 53 (15.7) | 166 (20.1) | 171 (19.3) | 101 (12.2)) | 105 (27.0) | 208 (32.2) | 226 (32.9) | 217 (29.4) | 224 (28.9) | 83 (28.2) | 87 (28.0) |
| ≥27 | 31 (9.4) | 38 (11.3) | 78 (9.1) | 95 (10.7) | 40 (10.7) | 49 (12.6) | 119 (18.4) | 155 (22.6)) | 129 (17.5) | 155 (20.0) | 56 (19.1) | 68 (21.9) |

**Table S4-1. Distribution of BMI values for interval changes in the LDL-C component in the cohort population (N=3,243)**

LDL-C, low density lipoprotein cholesterol; BMI, body mass index; TwSHHH, Taiwanese Survey on Prevalence of Hypertension, Hyperglycemia, and Hyperlipidemia.

**Table S4-2. BMI change between TwSHHH 2002 and TwSHHH 2007 for LDL-C component in the cohort population (N=3,410)**

†The sum of percentage does not equal to 100% in this column because of individuals with missing information (<1.0%) for this characteristic.

BMI, body mass index; TwSHHH, Taiwanese Survey on Prevalence of Hypertension, Hyperglycemia, and Hyperlipidemia; LDL-C, low density lipoprotein cholesterol.

|  | **BMI in TwSHHH 2002 (kg/m^2^)** | | | |
| --- | --- | --- | --- | --- |
|  | <18.5 | 18.5-23.9† | 24-26.9† | ≥27† |
| **BMI in TwSHHH 2007** **(kg/m^2^, %)** |  |  |  |  |
| <18.5 | 166 (83.4) | 26 (1.4) | 0 (0.0) | 0 (0.0) |
| 18.5-23.9 | 33 (16.6) | 1700 (91.6) | 59 (6.8) | 0 (0.0) |
| 24-26.9 | 0 (0.0) | 116 (6.3) | 719 (82.6) | 28 (5.8) |
| ≥27 | 0 (0.0) | 7 (0.4) | 86 (9.9) | 454 (93.8) |

|  | **Low-decreased** | **Low-stable** | **Low-increased** | **High-decreased** | **High-stable** | **High-increased** |
| --- | --- | --- | --- | --- | --- | --- |
| **Exclusion of cases with interval body weight loss**† |  |  |  |  |  |  |
| TC | 1 | 0.38 (0.21-0.67) | 0.38 (0.18-0.79) | 0.48 (0.27-0.87) | 0.43 (0.2-0.77) | 0.53 (0.25-1.10) |
| LDL-C | 1 | 0.67 (0.38-1.12) | 0.56 (0.29-1.11) | 0.73 (0.42-1.28) | 0.60 (0.34-1.05) | 0.66 (0.33-1.30) |
| Non-HDL-C | 1 | 0.84 (0.41-1.73) | 0.57 (0.24-1.34) | 0.79 (0.37-1.68) | 0.61 (0.29-1.27) | 0.73 (0.32-1.66) |

**Table S4-3. Sensitivity analysis for the adjusted relative risks and 95% confidence intervals of all-cause cancer incidence according to interval changes of each lipid component**

†Between Taiwanese Survey on Prevalence of Hypertension, Hyperglycemia, and Hyperlipidemia (TwSHHH) 2002 and TwSHHH 2007. LDL-C, low density lipoprotein cholesterol; TC, total cholesterol; Non-HDL-C, non-high-density lipoprotein cholesterol; Model adjusted for age, gender, body mass index, current smoking, alcohol drinking, betel nut consumption, regular exercise, marital status, education level, income level, diabetes mellitus, hypertension, high-sensitivity C-reactive protein, menopause status, hormone replacement therapy, and lipid-lowering agent use.
