# Supplementary material for: Lipid biomarkers and Cancer risk - a population-based prospective cohort study in Taiwan
Source: Lipids Health Dis. 2021 Oct 10;20:133. doi: 10.1186/s12944-021-01570-1 (PMC8502377; doi:10.1186/s12944-021-01570-1)
Supplement: Supplementary file 5 — Additional file 5: Table S5: Time distribution of cancer diagnosis after TwSHHH 2002 according to the interval changes in each lipid component. [file 12944_2021_1570_MOESM5_ESM.doc]

|  |  | **Characteristic of time interval between lipid content reduction and cancer diagnosis (years)** | | | | | |
| --- | --- | --- | --- | --- | --- | --- | --- |
| **Group** |  | **Mean** | **SD** | **Median** | **IQR (25^TH^, 75^TH^)** | **Maximum** | **Minimum** |
| **Low-decreased** | TC | 8.4 | 2.6 | 7.9 | (6.1, 10.0) | 12.9 | 5.4 |
|  | LDL-C | 8.7 | 2.5 | 7.9 | (6.5, 11.1) | 13.4 | 5.5 |
|  | TG† | - | - | - | - | - | - |
|  | Non-HDL-C | 8.4 | 2.0 | 7.7 | (7.2, 9.4) | 12.3 | 5.4 |
| **Low-stable** | TC | 9.4 | 2.5 | 9.3 | (7.0, 11.5) | 13.7 | 5.5 |
|  | LDL-C | 9.2 | 2.6 | 9.1 | (6.8, 11.8) | 13.7 | 5.4 |
|  | TG | 9.1 | 2.5 | 8.8 | (7.0, 11.1) | 13.7 | 5.4 |
|  | Non-HDL-C | 9.2 | 2.5 | 9.1 | (6.7, 11.1) | 13.7 | 5.5 |
| **Low-increased** | TC | 10.1 | 2.3 | 10.9 | (8.0, 12.0) | 13.4 | 6.0 |
|  | LDL-C | 10.6 | 2.1 | 10.6 | (9.0, 12.4) | 13.7 | 7.0 |
|  | TG | 9.9 | 2.3 | 10.4 | (8.8, 11.8) | 12.6 | 5.7 |
|  | Non-HDL-C | 10.2 | 2.3 | 10.5 | (8.3, 12.0) | 13.4 | 5.4 |
| **High-decreased** | TC | 9.8 | 2.6 | 9.7 | (7.4, 12.3) | 13.7 | 5.3 |
|  | LDL-C | 9.9 | 2.3 | 9.7 | (8.2, 11.9) | 13.6 | 5.6 |
|  | TG | 9.0 | 2.2 | 9.3 | (7.0, 10.4) | 12.9 | 5.3 |
|  | Non-HDL-C | 9.8 | 2.6 | 9.7 | (7.9, 12.3) | 13.7 | 5.3 |
| **High-stable** | TC | 9.8 | 2.3 | 10.2 | (7.6, 11.6) | 13.7 | 5.4 |
|  | LDL-C | 9.6 | 2.6 | 10.5 | (7.2, 11.6) | 13.7 | 5.3 |
|  | TG | 10.2 | 2.6 | 10.6 | (7.5, 12.4) | 13.7 | 5.5 |
|  | Non-HDL-C | 9.6 | 2.5 | 9.8 | (7.3, 11.6) | 13.6 | 5.7 |
| **High-increased** | TC | 9.8 | 2.5 | 10.4 | (7.7, 11.6) | 13.4 | 5.9 |
|  | LDL-C | 9.5 | 2.4 | 10.2 | (7.2, 11.0) | 13.3 | 5.8 |
|  | TG | 9.8 | 2.1 | 9.3 | (8.6, 11.8) | 13.4 | 6.1 |
|  | Non-HDL-C | 10.3 | 2.2 | 10.6 | (9.3, 11.6) | 13.7 | 5.8 |

**Table S5. Time distribution of cancer diagnosis after TwSHHH 2002 according to the interval changes in each lipid component**

†No cancer event reported in this row. TwSHHH, Taiwanese Survey on Prevalence of Hypertension, Hyperglycemia, and Hyperlipidemia; TC, total cholesterol; LDL-C, low density lipoprotein cholesterol; TG, triglycerides; Non-HDL-C, non-high-density lipoprotein cholesterol; SD, standard deviation; IQR, interquartile range.
